# Geometric morphometric analysis reveals age-related differences in the distal femur of Europeans

**DOI:** 10.1186/s40634-017-0095-3

**Published:** 2017-06-12

**Authors:** Etienne Cavaignac, Frederic Savall, Elodie Chantalat, Marie Faruch, Nicolas Reina, Philippe Chiron, Norbert Telmon

**Affiliations:** 1 Laboratoire AMIS, UMR 5288 CNRS, Université Paul Sabatier, 37 allée Jules Guesde, 31000 Toulouse, France; 2 Institut de l’appareil locomoteur, Hôpital Pierre-Paul Riquet, CHU Toulouse, France; 3 Laboratoire d’anatomie, Université Paul Sabatier, Toulouse, France

**Keywords:** Distal femur dimorphism, Principal component analysis, Procrustes analysis, Geometric morphometric analysis, Biological anthropology

## Abstract

**Background:**

Few studies have looked into age-related variations in femur shape. We hypothesized that three-dimensional (3D) geometric morphometric analysis of the distal femur would reveal age-related differences. The purpose of this study was to show that differences in distal femur shape related to age could be identified, visualized, and quantified using three-dimensional (3D) geometric morphometric analysis.

**Methods:**

Geometric morphometric analysis was carried out on CT scans of the distal femur of 256 subjects living in the south of France. Ten landmarks were defined on 3D reconstructions of the distal femur. Both traditional metric and geometric morphometric analyses were carried out on these bone reconstructions. These analyses were used to identify trends in bone shape in various age-based subgroups (<40, 40–60, >60).

**Results:**

Only the average bone shape of the < 40-year subgroup was statistically different from that of the other two groups. When the population was divided into two subgroups using 40 years of age as a threshold, the subject's age was correctly assigned 80% of the time.

**Discussion:**

Age-related differences are present in this bone segment. This reliable, accurate method could be used for virtual autopsy and to perform diachronic and interethnic comparisons. Moreover, this study provides updated morphometric data for a modern population in the south of France.

**Conclusion:**

Manufacturers of knee replacement implants will have to adapt their prosthesis models as the population evolves over time.

## Background

The sex of human remains can be determined by analyzing human bones (Ozer & Katayama 2008). The review of literature by Ozer et al. has shown that sex can be estimated using femoral dimorphism (Ozer & Katayama 2008). However, few studies have looked into age-related variations in femur shape (Barrier et al. 2009; Han et al. 2015). Age is typically determined using metric measurements between distinct points on the femur. (Han et al. 2015) However, these metric methods suffer from analysis bias related to inter- and intra-observer errors, observer experience, standardization challenges and problems related to statistical analysis (Gonzalez et al. 2009).

Geometric morphometric analysis can be used to quantify morphological features (Cavaignac et al. 2016). This technique allows the overall shape of an object to be analyzed with its geometry intact, making statistical analysis possible (Hennessy & Stringer 2002). It was developed to quantify the shape of rigid structures consisting of curves and bulges that are not easy to interpret using traditional metric methods (Bookstein 1978). This method has demonstrated its usefulness in physical anthropology (Bilfeld et al. 2015). To the best of our knowledge, this method has not been used to analyze the age-related differences in the distal femur. The distal femur is a rigid structure with curves and bulges so geometric morphometric analysis seems to be an appropriate method to explore it. With this method, the shape of two or more objects can be compared while disregarding the volume of these objects (Bilfeld et al. 2012). Since the size is normalized, the analysis can focus on the shape.

Age determination is a critical element of anthropology and forensic medicine (Barrier et al. 2009; Martrille et al. 2007). Several statistical models have been developed to determine person’s age using various bone fragments (Kim et al. 2013b). The femur is the longest bone and it is often well preserved (King et al. 1998; Slaus et al. 2003; Trancho et al. 1997). We believe it is relevant to analyze age variations in this bone with a method that can be used in both living and deceased subjects.

Bone shapes changes as a person ages (MacLatchy et al. 2000). We believe it is important to describe these changes in the shape of the distal femur, as the shape of the distal femur has a direct impact on the design of total knee replacement implants.

We hypothesized that three-dimensional (3D) geometric morphometric analysis of the distal femur would reveal age-related differences. The goal of this study was to show that differences in distal femur shape related age could be identified, visualized, and quantified using 3D geometric morphometric analysis.

## Methods

This was a retrospective descriptive analytical study. The research ethics committee at our healthcare facility approved this study (number 01–0415).

### Materials

#### Study population

**Fig. 1:**
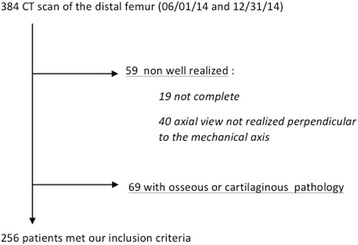
Flow chart of our studied population

Between June 1, 2014 and December 31, 2014, 256 CT scans of the distal femur met our inclusion criteria (Fig. 1). There were 134 women and 122 men. The average age was 58 ± 15.2 years. The right side was analyzed 122 times and the left side 134 times. The groups were comparable (Table 1). The analysis was carried out on the CT images of 256 distal femurs stored in our facility's imaging database. Only scans showing the entire distal femur (tip of femoral groove to most distal aspect of femur) without signs of disease conditions or osteoarthritis were retained. The included CT scans had mainly been performed to assess leg vasculature (CT angiogram) or to evaluate a tibial plateau fracture without previous history of knee problem and without lesions in the distal femur.

**Table 1:** Mean age of the various subgroups relative to sex and side. Comparisons were performed with student's *t*-test

		Age	*P*
Sex	Male (*n* = 134)	56.7 ± 14.42	0.445
	Female (*n* = 122)	58.14 ± 15.5	
Side	Right (*n* = 122)	57.36 ± 15.3	0.885
	Left (*n* = 134)	57.43 ± 14.7	

The CT scans were taken on a Sensation 16 Scanner (Siemens, Erlangen, Germany). Scanning was performed with the following parameters: 80 kV, 70 mA, gantry rotation time of 2 s, 144-mm table height, and axial scanning mode. The thickness of the reconstructed sections was kept constant at 2 mm every 1 mm. The image matrix was 512*512 pixels. A bone filter and a soft tissue filter were used.

The CT scans were saved as digital imaging and communications in medicine (DICOM) files and then processed with Amira 4.1.1® software (Mercury Computer System, Inc., Chelmsford, MA, USA).

### Methods

#### 3D morphological analysis

**Fig. 2:**
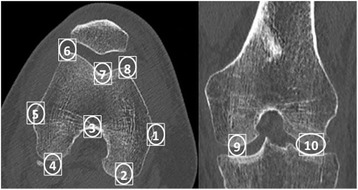
Location of landmarks on axial (left) and frontal (right) CT scan slices: 1) medial epicondyle, 2) most dorsal point on medial condyle, 3) top of intercondylar notch, 4) most dorsal point on lateral condyle, 5) lateral epicondyle, 6) most ventral point on lateral edge of trochlear groove, 7) most distal point at bottom of the trochlear groove, 8) most ventral point on the medial edge of the trochlear groove, 9) most distal point on medial condyle, 10) most distal point on lateral condyle

**Fig. 3:**
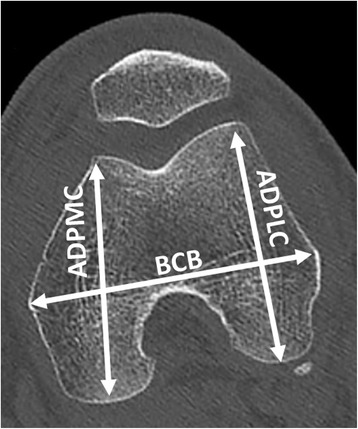
Osteometric data used to measure the plausibility of the study's methodology. EB: Epicondylar breadth, distance between the two epicondyles, APDMC: Anterior posterior diameter of the medial condyle, which is largest anteroposterior dimension of the medial condyle (Srivastava et al. 2012) and APDLC: Anterior posterior diameter of the lateral condyle, which is largest anteroposterior dimension of the lateral condyle

Ten osteometric landmarks were defined based on standard bone landmarks used in anthropometry (Fig. 2 and Table 2) (Bellemans et al. 2010). By using points typically associated with osteometric techniques, comparisons could be made with published studies to determine the plausibility of our results. The metric variables measured were the bicondylar breadth (BCB), which is the distance between the two epicondyles (Iscan & Shihai 1995),anterior posterior diameter of the medial condyle (APDMC), which is the largest anteroposterior dimension of the medial condyle (Srivastava et al. 2012), and anterior posterior diameter of the lateral condyle (APDLC), which is the largest anteroposterior dimension of the lateral condyle (Pinskerova et al. 2014) (Fig. 3). Once these landmarks had been located with 3D in vivo imaging software (Amira®, Visualization Sciences Group, Bordeaux, France), the coordinates of each landmark in space (x,y,z) were recorded.

**Table 2:** Anatomical description of the various landmarks used, with the intra- and interobserver variability for each. The error is given as a percentage

Landmark	Location	Intra-observer Variability	Inter-observer Variability
1	Medial epicondyle	1.64	1.63
2	Most dorsal point on medial condyle	1.64	1.64
3	Top of intercondylar notch	1.64	1.64
4	Most dorsal point on lateral condyle	1.64	1.64
5	Lateral epicondyle	1.63	1.64
6	Most outside point on trochlear groove	1.63	1.65
7	Most distal point at bottom of trochlear groove	1.64	1.65
8	Most ventral point on margin of trochlear groove	1.64	1.65
9	Most distal point on medial condyle	1.53	1.51
10	Most distal point on lateral condyle	1.52	1.49

**Fig. 4:**
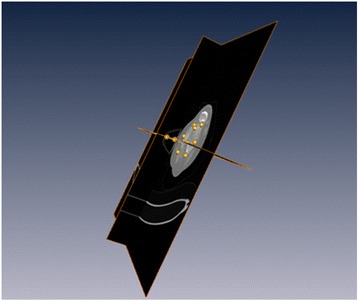
Creation of 3D reconstructions using the Amira 4.1.1® software (Mercury Computer System, Inc., Chelmsford, MA, USA). First, the axial plane in which the epicondyles were most prominent was identified. Reconstructions in the orthogonal planes were generated to position the landmarks

Axial slice where the epicondyles are more prominent were selected to place points 1–10. Oblique slices were created by resampling the images stack in order to be orthogonal to the axial plane (Fig. 4).

#### Reliability studies

The analyzed data were taken from the same database and analyzed twice on separate occasions by two observers. This made it possible to calculate the intra- and inter-observer variability for each landmark. For each observer, landmark deviations were calculated relative to the landmark mean value. The percentage error for each landmark was calculated, as described previously (von Cramon-Taubadel et al. 2007) (Table 2). The results were deemed acceptable if this error was less than 5% (von Cramon-Taubadel et al. 2007).

#### Procrustes analysis

All morphometric geometric analyses were carried out with Morpho J software (CP 2008) and R 2.2.0 software (Team 2014). The chosen landmarks made it possible to characterize the shape of the distal femur (Fig. 1). The first step consisted of a generalized Procrustes analysis (GPA) (Klingenberg 2002). As described previously (Bilfeld et al. 2013), this strategy expresses the results in graphical format by showing the average shape of the subgroups of interest.

### Statistical analysis

The descriptive analysis consisted of calculating the mean, median and standard deviation values for each subgroup. A comparative analysis was performed with all the variables based on age (<40, 40–60, > 60 years).

The landmark coordinates were analyzed using principal component analysis (PCA) (M Z 2004) and canonical variate analysis (CVA) to identify shape trends in the various subgroups (Bilfeld et al. 2013).

A discriminant analysis was performed to determine the percentage of cases in which the age was estimated correctly. Pearson’s Chi-square test was used to determine if this analysis was statistically significant (Elewa 2010). To determine if the difference between shapes was statistically significant, a *P*-value was also calculated using Goodall’s F-test and Mahalanobis D2 matrices (Oettle et al. 2009). The length variable (BCB) was compared using an analysis of variance (ANOVA).

## Results

### Reliability analysis

The percentage errors for the intra- and inter-observer comparisons for all the landmarks are given in Table 2 – none exceeded 2%.

### Age differences

**Fig. 5:**
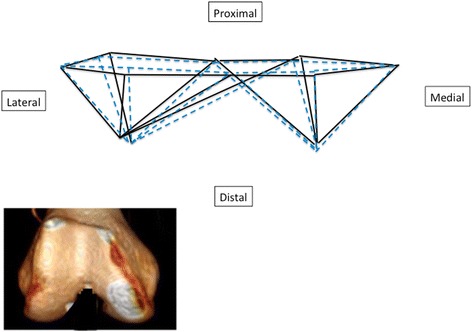
Shape variation based on age (>40: black solid line, < 40: blue dotted line). A 3D reconstruction is shown to make it easier to understand the data

**Fig. 6:**
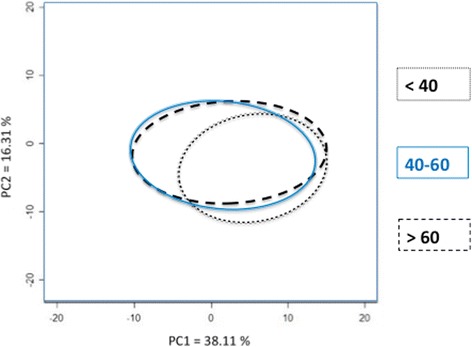
PCA obtained for the shape of the distal femur based on age. The ellipses correspond to 68% confidence intervals

The osteometric analysis (BCB, APDMC and APDLC) revealed no significant differences between the three subgroups of subjects (<40, 40–60, >60 years) (Table 3). Only the average bone shape of the < 40-year subgroup was statistically different from that of the other two groups (Table 4, Fig. 5). For the same femur size, < 40-year femurs are significantly longer in the frontal plane, i.e. the distance between the axial plane containing the epicondyles and the two most distal points on the condyles is greater in the < 40-year group. In the axial plane through the epicondyles, < 40-year femurs are shorter along the anteroposterior axis than > 40 year femurs, while the mediolateral distance is the same. The PCA based on age is shown in Fig. 6; principal component (PC)1 and PC2 accounted for 54.42% of the variance measured. When the population was divided into two subgroups using 40 years of age as a threshold, the subject's age was correctly assigned in 80% of the cases (original CVA) and in 74% of cases by cross-validated classification (Table 5).

**Table 3:** Mean values (± standard deviation) of the osteometric data for each subgroup based on age and sex. Comparisons were performed with an analysis of variance (ANOVA)

Age	<40	40–60	>60	*P*
BCB	80.3 ± 7.7	80.7 ± 6.6	80.4 ± 5.9	0.9
APDMC	62.8 ± 5.5	64.2 ± 5.4	63.5 ± 4.8	0.3
APDLC	62.7 ± 5.9	63 ± 4.9	62.6 ± 4.5	0.8

*BCB* BiCondylar breadth. *APDMC* Anterior posterior diameter of the medial condyle, *APDLC* Anterior posterior diameter of the lateral condyle

**Table 4:** Values of Goodall’s F and Mahalanobis D2 distance for the comparisons performed

Comparison	Mahalanobis D2 distance	Goodall’s F test	*P*
<40 vs. > 60	1.73	0.04	0.001
40–60 vs. > 60	0.68	0.019	0.78
<40 vs. 40–60	1.8	0.056	0.0002

**Table 5:** Results of the canonical variate analysis (CVA) and cross-validation for the age determination

	Original CVA			Cross-validated		
	Correctly assigned	Incorrectly assigned	% Correctly assigned	Correctly assigned	Incorrectly assigned	% Correctly assigned
<40	24	13	64.9	14	23	60.9
>40	182	37	83.1	176	43	80.4
Total	206	50	80	190	66	74

## Discussion

Our hypothesis is confirmed: 3D geometric morphometric analysis of the distal femur revealed differences between age groups (Fig. 5). Geometric morphometric analysis revealed age-related differences in the shape of the distal femur (Table 4). The shape of the femur in subjects under 40 years of age was different than the shape of the femur in older subjects. Classic osteometric analysis did not reveal age-related differences in the distal femur (Table 3). This means there are no differences in femur size between the three age groups, but for the same size of femur, the shape differs.

One of the main objectives of physical anthropology is to estimate a person's age and sex in the forensic or anthropology context (Barrier et al. 2009; Martrille et al. 2007). Most of the postcranial bones have been used to determine anthropological data of human remains through various statistical models (Kim et al. 2013b). The femur is the longest bone and it is often well preserved. As a consequence, we feel it is relevant to develop a method that can be used to determine a person’s age based on this bone (King et al. 1998; Slaus et al. 2003; Trancho et al. 1997) The large number of subjects (*n* = 256) included in this study has provided osteometric references related to age differences in a modern European population. Moreover, since this methodology can be used in living and deceased persons, it can be used in forensic medicine to determine age of a person in a legal context.

This is the first 3D study to show age-related differences in the overall shape of the distal femur, as the shape was different in subjects under 40 years of age and those over 40 years of age (Fig. 3). Discriminant analysis showed that 80% of subjects were correctly classified (original CVA). Although this method is not sufficiently accurate to be used alone, it can be used in the context of virtual or in vivo autopsy (Dedouit et al. 2015; Dedouit et al. 2014).

The age-related variations observed in the shape of the distal femur have consequences for orthopedic surgery, particularly for total knee arthroplasty (TKA). A better grasp of knee morphology and its variations can improve the design of TKA implants (Han et al. 2015). The same kind of implants are not suitable for different populations (Ho et al. 2006). Differences in shape have been reported by gender and ethnic groups (Bellemans et al. 2010). We are the first group to show differences in distal femur shape relative to age that are independent of the difference in size. In our study, we analyzed the differences in shape, not size. For these reasons, only adjusting the implant size does not solve the problem – the shape must be taken into account. Our study is the first to show age-related differences (<40 years and > 40 years) in a Caucasian population. The design of total knee arthroplasty implants is based on the anatomy of a Caucasian population (Mahfouz et al. 2012). Successful component placement in knee arthroplasty includes minimal overhang and good bone coverage (Bonnin et al. 2013). As a consequence, the age-related variations in a Caucasian population have to be take into account by manufacturers to modify the implant design over time.

Han et al. studied age-related anthropometric differences in Asians by analyzing MRI images of 535 knees. They used 20-year bands to evaluate successive generations. They found statistically significant differences in the classic anthropometric data between all the age bands. Although we also split our study population into 20-year segments, only the < 40-year population was significantly different to the others. This disparity can be explained by interethnic variability (Purkait & Chandra 2004). In addition, we performed a 3D analysis of the shape of the entire distal femur, while Han et al. performed two-dimensional analyses in various planes.

Our study is the most extensive up to now to evaluate age dimorphism of the distal femur in a modern European population. This data set can be used as a current reference when virtual or in vivo autopsy is performed (Dedouit et al. 2015; Dedouit et al. 2014). Temporal changes observed in modern populations mean that certain bone measurements must be re-evaluated over time (Alunni-Perret et al. 2008). Moreover, intergenerational variability must be taken into account when comparing populations (Han et al. 2015). Bias will be introduced into the analysis if the populations being compared are not from the same generation.

In our study, osteometric analyses were carried out in addition to the 3D analyses. By placing easily identifiable points on the apex of the bone contours, we obtained data in the traditional manner, which allowed us to verify that these data were consistent with published values (Table 6). The EB values reported by Han et al. (Han et al. 2015) were comparable to ours (Table 3) : group < 40 years, EB = 74.2 ± 2.1; group 40–60 years, EB = 73.4 ± 2.99 and group > 60 years, EB = 74.12 ± 3.24. Origin-based variability (Purkait & Chandra 2004) and sex-related variability must be taken into account when performing comparisons with published data, but the results of EB measurement are consistent (Table 6). Furthermore, the intra- and inter-observer error rates were very low in our study (Table 2). These two aspects (reproducibility and plausibility) validate our methodology. In addition, we only used femurs with no signs of bone pathology or osteoarthritis; any patients with osteoarthritis were excluded because this disease can alter the shape of the distal femur (Yip et al. 2004). Contrary to previous OA studies, we found that older patients had a smaller femur (Ding et al. 2005). Murshed et al. reported similar findings when analyzing femurs free of bone pathology (Murshed et al. 2005).

**Table 6:** Published osteometric data. Mean values with standard deviation

Nationality	BCB			ADPMC		ADPLC		n
	Female	Male	Ac.	Female	Male	Female	Male	
Spanish(Trancho et al. 1997)	70.8 ± 2.3	80.6 ± 2.9	97.5	NA	NA	NA	NA	132
French(Alunni-Perret et al. 2008)	74.8 ± 2.5	84.3 ± 3.6	95.4	NA	NA	NA	NA	88
Chinese(Iscan & Shihai 1995)	70.6 ± 3.2	80.3 ± 4.2	94.9	NA	NA	NA	NA	87
Thai (King et al. 1998)	75.4 ± 5.4	83.7 ± 4.7	93.3	NA	NA	NA	NA	104
North Indians(Srivastava et al. 2012)	68.3 ± 4	76.8 ± 4.2	85.1 (M) 78.6 (F)	54 ± 3.2	59.4 ± 3.3	55.6 ± 3.4	60.3 ± 3	122
Croatian(Slaus et al. 2003)	75.1 ± 3.3	86.7 ± 4.3	91.3	NA	NA	NA	NA	195
White South African (Steyn & Iscan 1997)	75.1 ± 3.3	84.6 ± 4.6	90.5	NA	NA	NA	NA	106
Indian(Purkait & Chandra 2004)	66.8 ± 4.2	78.7 ± 4.5	90.3	NA	NA	NA	NA	124
Chinese(Wu 1989)	69.3 ± 3	77.8 ± 5.8	83.7	NA	NA	NA	NA	141
German (Mall et al. 2000)	77 ± 5	84.0 ± 10	81.4	NA	NA	NA	NA	170
Czech (Pinskerova et al. 2014)	78.2	88.8	NA	65.6	71.8	63.4	69.9	200
Korean (Kim et al. 2013a)	NA	NA	NA	55.3 ± 3	61.2 ± 3	58.4 ± 2.8	64.6 ± 3	202
Our STUDY	75.5 ± 3.7	85.1 ± 4.9	88	60.4 ± 3.9	66.7 ± 4.2	60.4 ± 3.8	65.3 ± 4	255

*BCB* Bicondylar breadth, *APDMC* Anterior posterior diameter of the medial condyle, *APDLC* Anterior posterior diameter of the lateral condyle, *Ac* Accuracy is the percentage of correct assignment. *n* number of subjects in the study

Anthropometric data varies not only as a function of ethnicity, but also genetic, environmental, socioeconomic and nutritional factors (Han et al. 2015). Age-related variations may be related to the differences in height and weight between generations (Yoshiike et al. 2002).

The current study has certain limitations. Skeletally immature subjects were not included. In younger persons, the bone contours of the distal femoral epiphysis are not completely ossified. This would have increased the possibility of errors during landmark placement by the observers. In addition, very few subjects were under 40 years of age. Diseases that do not affect the distal femur but may require a CT scan that includes the distal femur, such as vascular conditions and tibial plateau fracture, are more common in older subjects. Furthermore, the age cut-off for the subgroups was chosen arbitrarily and not based on validated data, although we used previously described age brackets (Han et al. 2015). We analyzed the relationship between age and femur shape, not the changes during aging. A longitudinal study would be needed to measure changes in anthropological measurements as a person ages. While only the distal femur was analyzed in this study, it would be interesting to pair our analysis with data on the patients’ morphotype or other femur anatomy data. However, additional analyses could not be performed since the records were anonymized and the patients had no complaints related to their knee joint.

## Conclusion

The distal femur exhibits age-related differences. Three-dimensional geometric morphometric analysis made it possible to show these differences. Based on our findings, we feel that changes in bone anatomy over time cannot be ignored. It would be too simplistic to say that patients under 40 years of age require a different knee implant design because their distal femur differs in shape from older adults. TKA indications in patients under 40 years of age are extremely rare. Implant manufacturers must recognize that patient anatomy changes and that implant design should be reevaluated regularly.
